# Quantification of Humidity and Salt Detection in Historical Building Materials via Broadband Radar Measurement

**DOI:** 10.3390/s23104616

**Published:** 2023-05-10

**Authors:** Oliver Blaschke, Felix Brand, Klaus Stefan Drese

**Affiliations:** Institute of Sensor and Actuator Technology, Coburg University of Applied Sciences and Arts, Am Hofbräuhaus 1b, 96450 Coburg, Germany; felix.brand@hs-coburg.de (F.B.); klaus.drese@hs-coburg.de (K.S.D.)

**Keywords:** moisture, ground penetrating radar, noninvasive measurement, moisture measurement, masonry, salinity, water content

## Abstract

For the investigation of moisture and salt content in historic masonry, destructive drilling samples followed by a gravimetric investigation is still the preferred method. In order to prevent the destructive intrusion into the building substance and to enable a large-area measurement, a nondestructive and easy-to-use measuring principle is needed. Previous systems for moisture measurement usually fail due to a strong dependence on contained salts. In this work, a ground penetrating radar (GPR) system was used to determine the frequency-dependent complex permittivity in the range between 1 and 3 GHz on salt-loaded samples of historical building materials. By choosing this frequency range, it was possible to determine the moisture in the samples independently of the salt content. In addition, it was possible to make a quantitative statement about the salt level. The applied method demonstrates that with ground penetrating radar measurements in the frequency range selected here, a salt-independent moisture determination can be carried out.

## 1. Introduction

High levels of salt and moisture in masonry can cause significant damage. Alongside aesthetic impairments (discoloration, salt crystallization, crumbling of plaster and paint), high moisture contents can result in structural problems (decrease in compressive strength of materials) as well as worsening the indoor climate (fungal attack, degradation of thermal insulation) [1,2]. Therefore, it is important to measure and control the varying moisture content regularly in order to evaluate the risk of damage and to take preventive conservation measures [2].

Special attention has to be paid to the water and salt contents during the restoration and maintenance of historic buildings, which often lack horizontal water spears and are therefore susceptible to moisture [1,3].

Detailed knowledge of the actual condition of the masonry enables targeted and gentle treatment to prevent further damage and maintain the original building structure. Likewise, early detection of penetrating moisture and rapid treatment can prevent damage.

To address these problems, a measuring method is needed that can nondestructively detect the moisture and salt content of masonry walls, and it should be easy and fast to handle and operate.

A wide variety of methods to measure moisture in historical building materials such as sandstone and brick have been compiled in the literature over the past years. Techniques such as infrared thermography [4,5,6,7], high-frequency sensors [8,9], and evanescent field dielectrometry [10] have to be mentioned here. The latter has also been used for concrete [11,12]. Additionally, concrete and other construction materials, such as limestone, cement, clay, or other porous materials, were measured in the laboratory by gamma ray attenuation [13,14,15], X-ray radiography [16,17,18], neutron radiography [19,20,21,22], time-domain reflectometry [23,24,25], wireless inductive–capacitive sensors [26,27], optic fiber sensors [28,29,30,31,32], impedance tomography [33,34], and the capacitance method [35,36,37]. These methods are either too complex, too expensive, do not achieve the necessary resolution, are very susceptible to external influences, or are not available as a generally applicable measuring technique. Therefore, in the restoration and inspection of masonry, damaging drilling samples is still the standard for determining moisture and salt content.

The most promising method is the determination of the complex permittivity [38]. An overview of previous scientific works on this method is presented in the next chapter.

The aim of the study is to prove whether a quantitative salt and moisture determination is possible in a nondestructive manner with the help of a GPR sensor. Unlike previous studies, this study differs in the frequency-resolved broadband signal evaluation considering various relevant salts in historic masonry. In addition, the method presented here is capable of directly measuring on masonry without preparation or destructive sampling, in contrast to other studies using dielectric cells for broadband evaluation of radar signals.

Within this study it is shown that the moisture content of sandstones and bricks can be determined nondestructively by measuring the complex permittivity in the frequency range between 1 and 3 GHz. In addition, making a quantitative statement about existing dissolved salts is possible. The permittivity is measured using an stepped-frequency continuous wave (SFCW) ground penetrating radar (GPR) sensor based on the principle of time-domain reflectometry.

## 2. Materials and Methods

### 2.1. Theoretical Background

As mentioned above, the measurement of complex permittivity is a suitable parameter for investigating bound water in masonry. Free water is clearly different from bound water. The relaxation frequency for free water is about 16 GHz [38]. In 1974 [39], it was shown that the relaxation frequency of bound water in sand, silt, and clay is reduced to frequencies between 1 and 4 GHz. Later, this reduction in relaxation frequency was also demonstrated for concrete [40]. For historic building materials such as sandstone and brick, frequency-dependent dielectric constant values were measured in publications, but the relaxation frequency of the bound water could not be determined due to the limited frequency spectrum range of the measuring instruments [41,42,43]. However, based on the existing dielectric constant measurement values, it can be assumed that the relaxation frequency of the water is also greatly reduced in the later materials.

Previous scientific work determining the moisture content of bricks, sandstone, or limestone using radar waves is summarized in Table 1. These results can basically be divided into two different categories based on their measurement principle. The first group [42,43] used a dielectric cell to measure permittivity. For this purpose, a test specimen (70 mm × Ø 75 mm [41], 80–120 mm × Ø 102 mm (inner hole 45 mm) [43]) is inserted into a cell and the complex permittivity is measured as a function of frequency. West et al. [43] showed a quadratic relationship between the real part of the permittivity and the volumetric humidity using two frequencies (150 MHz, 500 MHz). In addition, it was found that clay and NaCl concentration have an influence on the real and imaginary parts of the electric permitivity. Gilbert et al. [42], on the other hand, chose an artificial intelligence (AI) model for evaluation that uses the frequency-dependent permittivity (50–300 MHz) as input variables. These results had a mean relative error of 1.9 vol%.

The second group of papers [42,44,45] used a pulse or stepped-frequency continuous wave ground penetrating radar (GPR) system to detect the temporal signal of the reflection from the back of the wall. This is a nondestructive measurement method that does not require sampling.

In the work of Agliata et al. [45], tuff bricks with an step frequency continuous wave ground penetrating radar (SFCW-GPR) were investigated. In the frequency range of 1.2 and 3.0 GHz, a constant permittivity as well as a constant electrical conductivity was assumed. Based on these assumptions, an inverse modeling of the radar signal allowed the calculation of both quantities. It was shown that both values correlated with the volumetric humidity in the range between 5 and 35 V%. Cetrangolo et al. [41] used a pulse radar with a center frequency of 1.6 GHz. The real part of the permittivity is determined on the basis of the transit time of the radar pulse. The permittivity determined in this way has a linear relationship with the volumetric moisture content. The correlation varies with different bricks.

Maierhofer [44] used a pulse radar with a very high frequency compared to other investigations (center frequency of 7.2 GHz and a 3 dB bandwidth of 0.325 GHz). The reason given for the high frequency is that the salt influence decreases with higher frequencies. The real part of the permittivity was determined by the transit time of the radar pulse and the imaginary part by the ratio of two intensities. Both quantities were not frequency-resolved. The building materials investigated were solid bricks, hollow bricks, and sand–lime bricks. The results show that the permittivity differs depending on the volumetric moisture of the building materials. The different salt loads (0.2–5.0 m% NaCl in water) are not noticeable in the real part of the permittivity, and in the imaginary part only at high NaCl concentration (5.0 m%).

**Table 1:** Overview of scientific studies on the determination of the moisture content in bricks, sandstone, and limestone using radar waves.

Source	Investigated Materials	Method	Measured Value	Frequency Range	Water Content Range	Quality of Results/Measurement Error
[46]	One type of saline brick	Dielectric and microwave method	Unspecified electric measurement	Unspecified	0–11 m% with different salt types and concentrations	10% mean relative error
[42]	One type of limestone	Dielectric cell	$\epsilon^{\prime }\left(f\right)$, $\epsilon^{\prime \prime }\left(f\right)$	0.05–0.3 GHz	0–100% saturation	1.9% mean relative error (saturation)
[43]	Different types of sandstone	Dielectric cell	$\epsilon^{\prime }\left(f\right)$, $\epsilon^{\prime \prime }\left(f\right)$	0.075–1 GHz	0–35 vol% with different clay and NaCl contents	8% relative error (estimated by author)
[45]	One type of brick	SFCW-GPR	Electrical conductivity, $\epsilon^{\prime }$	1.2–3 GHz	5–35 vol%	Not mentioned by author, estimated mean error: 3–4 vol%
[41]	Different types of ceramic bricks	Pulse-GPR	Time of flight, $\epsilon^{\prime }\left(1.6\;\mathrm{GHz}\right)$	1.6 GHz center frequency	0–30 vol%	Not mentioned by author, estimated mean relative error: 5 vol%
[44]	Different types of bricks	Pulse-GPR	$\epsilon^{\prime }\left(7.2\;\mathrm{GHz}\right)$, $\epsilon^{\prime \prime }\left(7.2\;\mathrm{GHz}\right)$	7.2 GHz center frequency	0–25 vol% with different NaCl content	Not mentioned by author

Apart from moisture analysis in natural stones and bricks, there are many studies on concrete [47,48,49,50,51,52,53,54,55]. Since this work focuses on historical building materials, concrete will not be discussed in detail here.

Based on the publications listed, it can be concluded that a high-frequency radar measurement is necessary to determine the moisture content of the building materials independently of the salts. Low-frequency radar signals can be used to obtain information on the salt content. Accordingly, the aim should be a radar measurement with the widest possible bandwidth and a frequency-resolved determination of the complex permittivity. In addition, it must be considered that the damping increases at higher frequencies. A dielectric cell should be avoided because of the destructive sampling while GPR systems allow nondestructive, uncomplicated, and large-area measurement of masonry.

For these reasons, the SFCW-GPR described in Section 2.2 was chosen, which has the highest bandwidth of commercially available GPR systems to our knowledge.

### 2.2. Materials and Samples

Measurements were carried out on two types of construction materials. The first one was brick, which was produced based on a historical manufacturing process (Wienerberger solid brick, NZ-Voll 2,0/20, 240 mm × 115 mm × 71 mm, 5 different samples). The second material was sandstone (Sander reed sandstone, 400 mm × 250 mm × 200 mm, 6 different samples). Both materials were chosen since they were used in historical buildings. Measurements were carried out along the 240 mm respectively 200 mm side. The physical properties of the samples are listed in Table 2.

The influence of NaCl (CAS: 7647-14-5), MgSO_4_ (CAS: 10034-99-8), and KNO_3_ (CAS: 7757-79-1) on the radar measurements were investigated.

The measurements were carried out using the commercial radar system GP 8800 from Screening Eagle. This system is a stepped-frequency continuous wave GPR with a frequency between 400 and 6000 MHz with the dimensions of 89 mm × 89 mm × 76 mm [56].

The scale used to measure the weight of the specimens had a sensitivity of 5 g.

**Figure 1:**
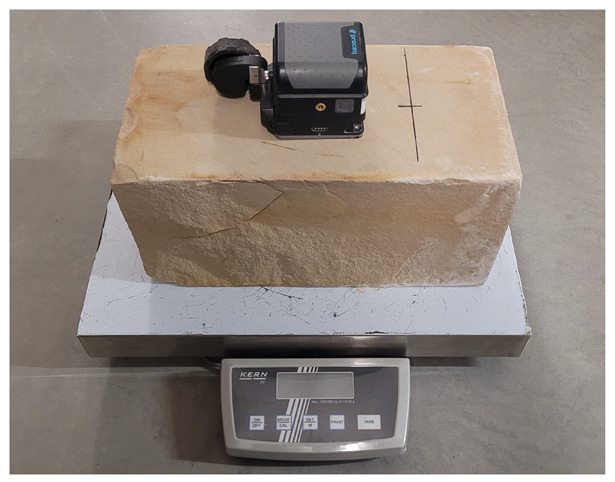
Measurement of a sandstone with the GP 8800 radar system.

The measurement equipment can be seen in Figure 1.

### 2.3. Preparation and Testing

To measure different moisture, we placed the specimens into a deionised water bath for a couple of days until the specimens were fully saturated. The drying of the stones was accompanied by measuring the reflection of the radar signals at the back of the stone. The radar probe was positioned at the center of the sample. To ensure that there were no additional interfering signals, the stone lay on a metal plate. Concurrently, the weight of the stone was measured to calculate the water content *w* inside the specimen as mass percent (m%).

(1) $$w=\frac{m_{meas}-m_{dry}}{m_{dry}}\times 100\%$$

The measurements were carried out twice per day until the samples reached a moisture equilibrium at room conditions. In addition, we evaluated the brick specimens after drying for two days at 105 ${}^{\circ }C$.

To measure different salt contents inside the material, the whole process was repeated using salt water instead of deionised water. The salt content in the water was adjusted to achieve an anion (Cl^−^, SO_4_^2−^, NO_3_^−^) concentration inside the dry sample of 0.1 m%. For this purpose, the soaking ratio mentioned in Table 2 was used.

### 2.4. Radar Data Processing

The calculation of the frequency-dependent phase velocity of the radar wave was performed with the phase spectral analysis [57]. This method needs a reference wave ref(t), which was prerecorded. The reference wave enables the calculation of the cross-power spectrum density (CPSD) to determine the frequency-dependent power of the reflected wave meas(t).

(2) $$CPSD=\mathrm{FFT}{\left(ref\right)}^{*}\times \mathrm{FFT}\left(meas\right)$$

with FFT being the fast Fourier transformation functional of the reference and measured time signal, and ${}^{*}$ indicating the complex conjugation.

By comparing the reference wave and the received wave, we can calculate the phase spectrum

(3) $$\phi =\frac{2\pi f}{\phi_{meas}\left(f\right)-\phi_{ref}\left(f\right)}$$

With ϕ being the phase of the reference/measured wave obtained by the fast Fourier transformation and *f* being the frequency.

Knowing the thickness of the specimens, Δx, the frequency dependent phase velocity can be calculated [58]:

(4) $$c_{ph}=\frac{2\pi f\Delta x}{\phi_{meas}\left(f\right)-\phi_{ref}\left(f\right)}$$

Using the Kramers–Kronig relation for nonmagnetic materials ($\mu_{r}\approx 1$), the complex permittivity $\epsilon =\epsilon^{\prime }+i\epsilon^{\prime \prime }$ can be calculated [59]:

(5) $$\begin{array}{c} \begin{array}{cc} {\left(n+ik\right)}^{2} & =\left(\epsilon^{\prime }+i\epsilon^{\prime \prime }\right)\mu_{r}\\ \epsilon^{\prime } & =n^{2}-k^{2}\\ \epsilon^{\prime \prime } & =2nk \end{array} \end{array}$$

For building materials, the imaginary refractive index *k* is usually small compared to the refractive index *n*. Additionally, the absorption coefficient can be approximated using the amplitude spectrum *A* of the reference and measured wave, with $c_{0}$ being the speed of light in a vacuum.

(6) $$\begin{array}{c} \begin{array}{cc} \epsilon^{\prime }\left(f\right) & \approx n^{2}={\left(\frac{c_{0}}{c_{ph}\left(f\right)}\right)}^{2}\\ \epsilon^{\prime \prime }\left(f\right) & \approx \ln\left(\frac{A_{ref}\left(f\right)}{A_{meas}\left(f\right)}\right)\frac{c_{0}}{\pi f\Delta x} \end{array} \end{array}$$

## 3. Results

### 3.1. Frequency-Dependent Effects

**Figure 2:**
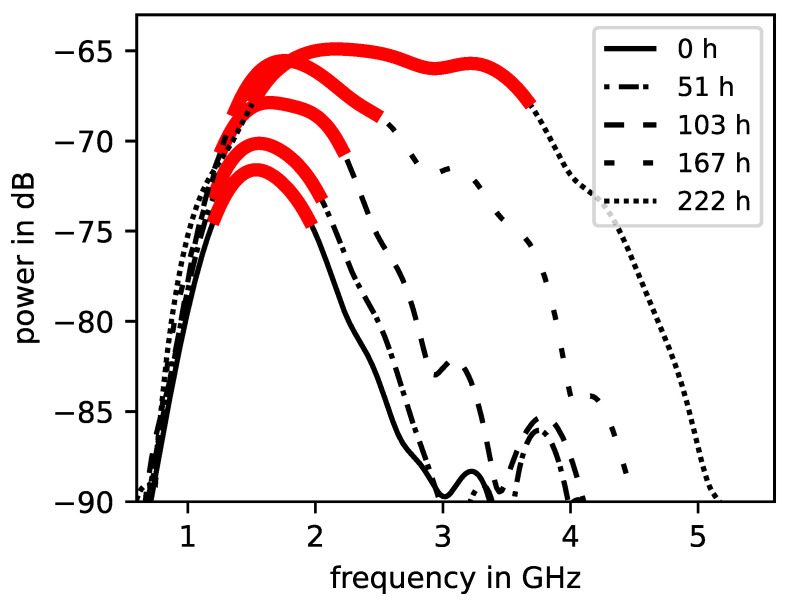
Cross-power density spectra as defined in Equation (2) from an initially saturated brick specimen at different drying times in hours according to the legend. Peak frequency range ( 3 dB drop) is marked in red/bold.

The measurements, carried out as stated in Section 2.3, were processed in such a way that at first the cross-power density spectra (CPSD) were calculated similar to in Equation (2). The CPSD for a single brick, which was initially completely saturated with deionised water, for different timestamps at the continuous drying process are shown in Figure 2. For every CPSD, there was a peak frequency range which was defined as the section which contained the 3 dB drop from the maximum value of the curve. The further evaluation of the signals in the frequency domain was restricted to this peak frequency range in order to maintain a significant signal intensity. Note that this peak frequency range broadens and also shifts to higher frequency components as the drying process continues. Further, there was an increase in total noticeable signal intensity. In fact, the higher frequency components are highly dampened in the initial drying process. Worth mentioning is that the frequency response of the CPSD is highly dependent on the characteristics of the SFCW radar device as the emitted frequency spectra, directivity, and the transmitted power varies. The signal intrusions, which are observable in Figure 2, lead from these device characteristics in combination with the attenuation of the construction material.

**Figure 3:**
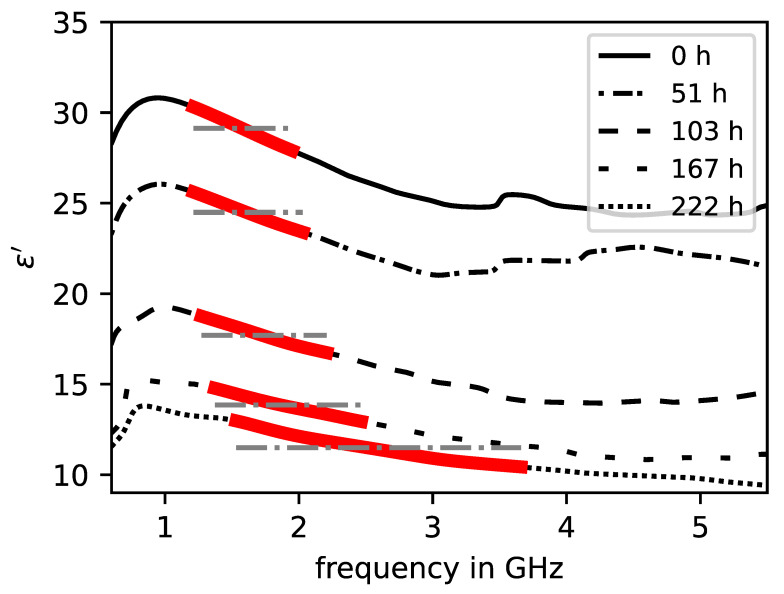
Dispersion curves of the real part of permittivity of an initially saturated brick specimen at different drying times. Significant evaluation range is marked in red with corresponding weighted mean model.

**Figure 4:**
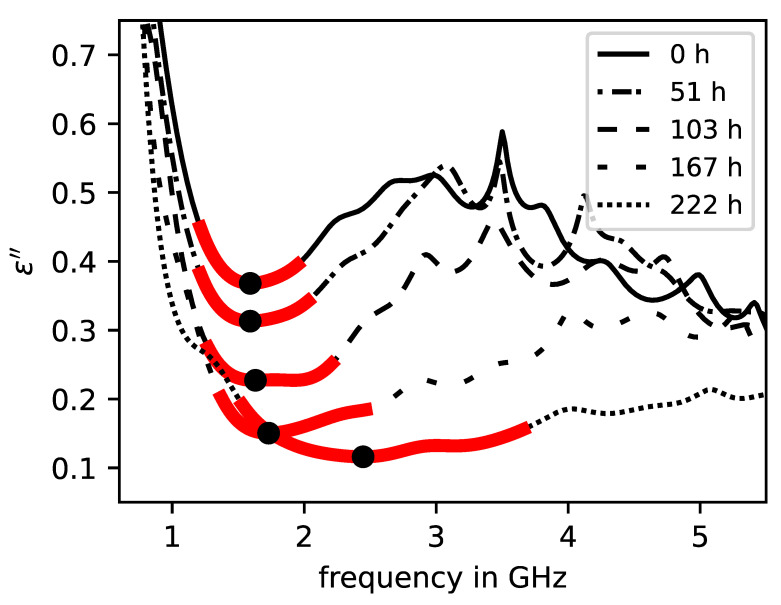
Dispersion curves of the imaginary part of permittivity of an initially saturated brick specimen at different drying times. Local extreme values (black dots) within the evaluation range (red lines) are model parameters.

The dispersion curves of the real and imaginary part of the complex relative permittivity associated with the above CPSD curves are displayed in Figure 3 and Figure 4. Similar to the CPSD, the permittivity curves are highly dependent on frequency and drying time, respectively, water content. In Figure 3, it is shown that the overall absolute value of the real part of permittivity in the peak frequency range decreases with increase in drying time and frequency. As for the imaginary part of the permittivity in Figure 4, a steep decline at the lower end of the frequency band can be observed for all curves at all drying times. With increase in frequency, the imaginary permittivity raises after a local minimum with a gradient dependent on the drying time. Similar to the real permittivity curves, the absolute values of the imaginary curves also decrease with the drying time.

To link the dispersion curves with the associated moisture determined by gravimetric analysis, various model curves were adapted to the curves of the real and imaginary part of the permittivity. For the real part, in addition to a constant model averaged over the peak frequency range, a linear model and a mean value model weighted with the normalized and logarithmic-scaled CPSD$w_{CPDS}$ were analyzed. The latter proved to be the most suitable and is defined as follows:

(7) $${\bar{\epsilon^{\prime }}}_{weight}=\frac{\sum \epsilon^{\prime }\left(f\right)\times w_{CPDS}\left(f\right)}{\sum w_{CPDS}\left(f\right)}.$$

The information of the imaginary part is represented by the absolute value of the local minimum in the peak frequency range. Figure 3 and Figure 4 show the curve sections of the real and imaginary part of the permittivity with adjusted parameters.

**Figure 5:**
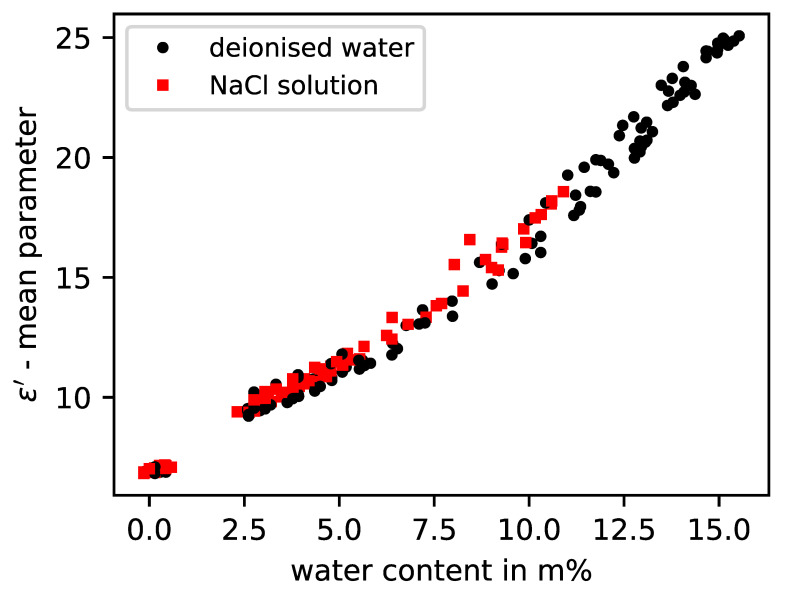
Real permittivity model values from five different brick specimens with either deionised water or NaCl solution according to measured water content.

**Figure 6:**
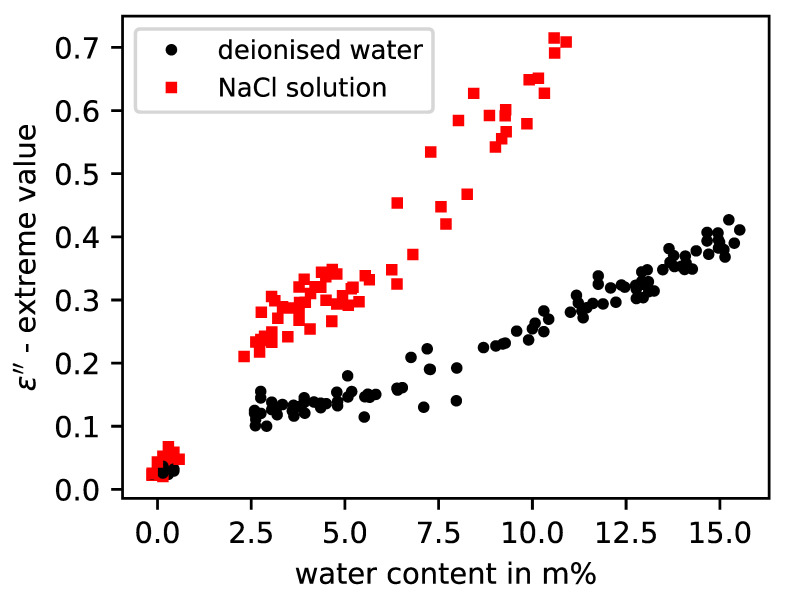
Imaginary permittivity model values from five different brick specimens with either deionised water or NaCl solution according to measured water content.

In Figure 5, it is shown that the parameters of the real part of the permittivity can represent the humidity independently of the salt content. The reason is the diminishing influence of the dissolved ions of the salts with increase in frequency. However, the imaginary part parameter shows a high sensitivity to the salt load in the examined frequency range, as depicted in Figure 6. By choosing suitable model parameters in conjunction with the moisture data obtained by weighing, it is possible to establish a link between the measured curves and the moisture, as can be seen for the building material brick in Figure 5 and Figure 6.

### 3.2. Salt-Dependent Effects

**Figure 7:**
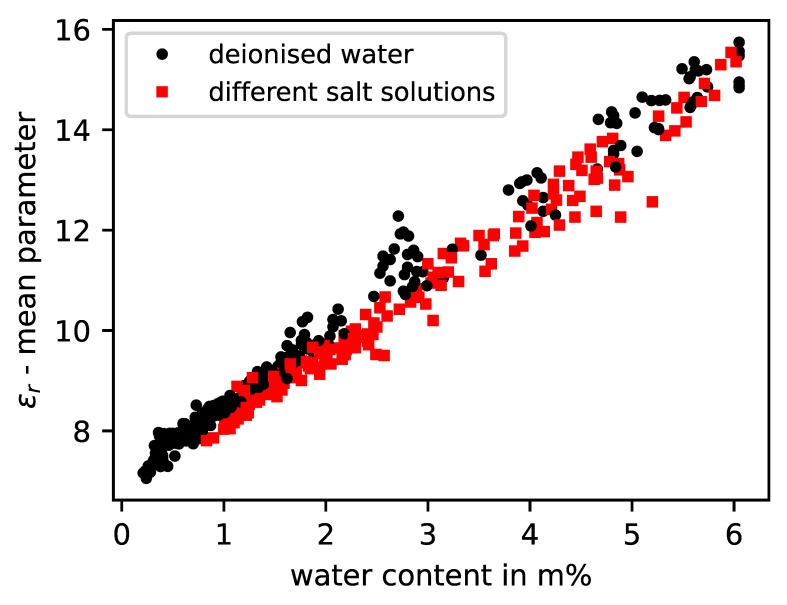
Real permittivity model values of six sandstone specimens with different salt solutions and deionised water according to measured water content.

**Figure 8:**
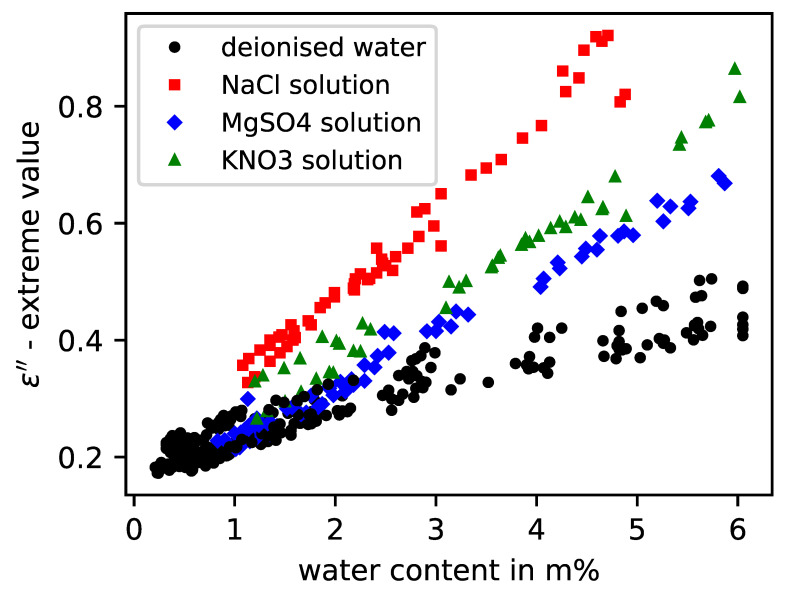
Imaginary permittivity model values of six sandstone specimens with different salt solutions and deionised water according to measured water content.

When applying the evaluation scheme used in this study to sandstone samples to which different types of structurally relevant salts were added, it was observed that again there was no significant impact of the salt on the real part of permittivity according to Figure 7. However, it was noticeable that different measurement effects can be noted in the imaginary part of the permittivity at the same anion concentration in mass percentage, which was 0.1 m% related to the dry sandstone samples. Figure 8 shows that sodium chloride at the same concentration has the strongest influence on the measurement signal and, thus, on the parameterized imaginary part absolute value. Over the considered moisture horizon above 1.0 m%, these values are clearly above the reference values of the samples mixed with deionised water, as well as the measured values of the other salt types. The samples with potassium nitrate and magnesium sulfate showed a medium attenuation. Basically, it can be stated that different salt types with the same total mass of anions cause different impacts on the measurement method related to the imaginary part value. At the current state, the presented method allows only a qualitative distinction between high, medium, and small attenuation effects caused by the dissolved salts in the specimens. A differentiation between diverse salt types cannot be performed, nor is it possible to measure the salts if they are in a crystalline state, as they are electrically neutral. This can be observed in Figure 8, as the measurements with saline specimens converge into the measurements with deionised water as the moisture level tends to zero.

### 3.3. Material Dependent Effects

**Figure 9:**
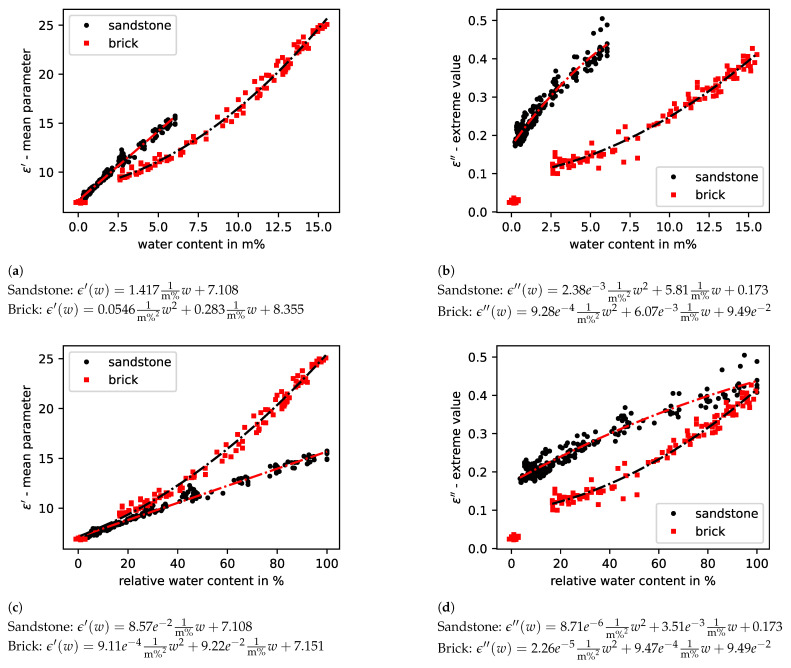
Model values of real (**a**,**c**) and imaginary (**b**,**d**) permittivity for brick and sandstone probes saturated with deionised water. Panels (**a**,**b**) depict the dependency on the absolute water content; (**c**,**d**) are scaled to the relative water content.

Investigations of bricks and sandstones showed that the different types of material cause different behavior in the absolute values and the slopes of the models. This is illustrated by Figure 9a–d for the real part, as well as the imaginary part, parameter of the complex permittivity.

It should be noted that the sandstones absorbed significantly less water than the bricks in relation to their dry mass. This results in a higher slope for the building material sandstone with a linear correlation between moisture and real part parameter value. The measurements on bricks have a parabolic dependence with deviation in the oven-dry measurements close to a moisture content of 0 m%. The imaginary part parameter is also clearly different, whereby the measurement points for sandstones with deionised water follow a root function-like course and the brick measurement points are mostly quadratic with the exception of the oven-dry measurement points, which were not used for fitting the model in Figure 9.

The root-mean-square deviation from the fitting curve in Figure 9a for brick is 0.99 m%, and for sandstone 0.19 m%, resulting in a relative error regarding the saturation humidity of 6.4% and 3.1%, respectively.

## 4. Discussion and Conclusions

The measurement method described here uses a variable frequency range, which is based on the signal intensity of the cross-power spectrum as a function of the moisture content of the examined historical building material. Due to the selected measuring range of above 1 GHz, a salt-independent moisture determination can be carried out within a single measurement, accompanied by the possibility of salt detection.

It is shown that at the same mass concentration of anions, different structurally relevant salts cause various measurement effects. Furthermore, only the dissolved salts can be detected with the measuring method, since in crystallized form the salt crystals are electrically neutral. A quantitative measurement of the salt content is therefore not possible, but at least a qualitative statement can be made. However, it is assumed that the attenuation effect of different salt types is mostly affected by the total amount of free ions, as sodium chloride has a much smaller molar mass as compared to potassium nitrate and magnesium sulfate. This topic is currently under research.

The examination of various historical building materials has shown that they differ in terms of measurement. A material-independent moisture measurement is therefore not feasible. However, prior referencing of the building materials to be examined under laboratory conditions can create a basis for determining the moisture in masonry in a nondestructive manner under real conditions. Alternatively, at least a relative observation of the permittivity can identify areas of different moisture content in a structure. These can be verified by targeted drilling sampling.

Further limitations of the current setup are a limited penetration depth of the radar signal resulting in a maximum specimen thickness of about 20 to 50 cm, which is highly dependent on the humidity and salt load. In addition, the measured moisture is a mean value across the sample thickness and does not allow the depth-resolved evaluation, except that there are different reflection positions inside the specimen, such as, e.g., multilayered walls.

The radar measurements allow qualitative statements to be made about the salt load, but do not allow the respective salts to be assigned. Similar to moisture and unknown masonry, a locally resolved measurement is comparative and enables the targeted drilling sampling for a detailed analysis of the occurring salts.

As for now, the error analyses show that simple polynomial models achieve a relative error for the investigated materials in the range of 3.1% (0.99 m%) and 6.4% (0.19 m%) independent of any added salts. Compared to the works mentioned in Table 1, the method presented here has a lower relative error. The exception is source [42], which does not use a GPR probe but, instead, uses a dielectric cell, which is a destructive measurement technique.

The further steps in optimizing the presented method would be to make use of machine learning algorithms to take more dispersion characteristics into account, as it was presented here with a weighted mean model and the local minimum model for the real and imaginary part of relative permittivity. In addition to the frequency domain data, machine learning algorithms can also make use of time-domain characteristics such as position of zero crossing of the backpropagated signal. The inclusion of these factors can result in an accurate prediction of moisture content and salt detection as well as a reduced expense in user interaction.

Thus, this method can replace, or at least reduce, the taking of drilling samples to a minimum in the case of known materials. The intervention in the building fabric is consequently reduced.

## Figures and Tables

**Table 2:** Overview of the physical properties of the used materials.

Material	Density	Porosity	Soaking Ratio
Brick	1.84 g/cm^3^	28.2%	15.6 m%
Sandstone	2.01 g/cm^3^	23.7%	6.1 m%

## Data Availability

The data presented in this study are available on request from the corresponding author.

## Abbreviations

The following abbreviations are used in this manuscript:

GPR	Ground penetrating radar
SFCW	Stepped-frequency continuous wave
CPSD	Cross-power spectrum density
